# Supplementary material for: Gradual compaction of the central spindle decreases its dynamicity in PRC1 and EB1 gene-edited cells
Source: Life Sci Alliance. 2021 Sep 27;4(12):e202101222. doi: 10.26508/lsa.202101222 (PMC8500333; doi:10.26508/lsa.202101222)
Supplement: Supplementary file 2 [file LSA-2021-01222_TableS1.docx]

Table S1. Summary of cell lines used in this study.

| hTERT-RPE1 cell lines | Clone number | Main Figures | Suppl. Figures | Suppl. Movies |
| --- | --- | --- | --- | --- |
| mGFP-EB1 (endogenous) | A3 | Figure 1 | Suppl. Figure 2 | Suppl. Movie 1 |
| mGFP-EB1 (endogenous)  & mCherry-PRC1 (endogenous) | B1 | Figure 1 | Suppl. Figures 2, 4 | Suppl. Movies 2, 3 |
| mCherry-PRC1 (endogenous) | C3 |  | Suppl. Figure 3 |  |
| mCherry-PRC1 (endogenous)  & EB1-GFP (ectopic) | D1 |  | Suppl. Figure 3 |  |
| mGFP-PRC1 (endogenous) | E1 | Figures 2, 3 | Suppl. Figures 5, 6 | Suppl. Movies 4, 5 |
| mCherry-PRC1 (endogenous)  & KIF4A-mGFP (ectopic) |  | Figure 4 |  | Suppl. Movie 6 |
| mCherry-PRC1 (endogenous)  & EGFP-CLASP1 (ectopic) |  | Figure 5 |  |  |
| unmodified control |  |  | Suppl. Figures 1, 2, 3, 5 |  |
