# Supplementary material for: Gradual compaction of the central spindle decreases its dynamicity in PRC1 and EB1 gene-edited cells
Source: Life Sci Alliance. 2021 Sep 27;4(12):e202101222. doi: 10.26508/lsa.202101222 (PMC8500333; doi:10.26508/lsa.202101222)
Supplement: Supplementary file 8 [file LSA-2021-01222_Supplemental_Data_1.pdf]

PRC1 donor vector primers

LHA(PRC1-N)FP	TCGGTACCCGGGGATCGAGCAAAGATGTTTAGAAGTACAATAG
LHA(PRC1-N)RPGFP	CCTTGCTCACGCTCATGGCGGACGCTCCAAGCAG
LHA(PRC1-N) RPmcherry	CGCCCTTGCTCACCATGGCGGACGCTCCAAGCAG
mGFP (PRC1-N)FP	ATGAGCGTGAGCAAGGGCGA
mGFP(PRC1-N)RP	CGCACCTTCTCCTCATTCCGCCTCCTCCGCCCGCGTATCCGCCTCCCTTG
Mcherry(PRC1-N)FP	ATGGTGAGCAAGGGCGAGGA
Mcherry(PRC1-N)RP	CGCACCTTCTCCTCATTCCGCCTCCTCCGCCCTTGTACAGCTCGTCCATGCC
RHA(PRC1-N)FP	ATGAGGAGAAGGTGCGGGTTG
RHA(PRC1-N)RP	TCGACTCTAGAGGATCTTGAGACGTAGTCTCACTCTGTC

SDM primers for the PAM motifs

SDMguideA FP	TTGTTGCTCTCGGGGGGGTGTGGAGTAGGTCT
SDMguideA RP	AGACCTACTCCACACCCCCCCGAGAGCAACAA
SDMguideB FP	AGCGTCCGCCATGAGAAGAAGGTGCGGGTT
SDMguideB RP	AACCCGCACCTTCTTCTCATGGCGGACGCT
SDMguideC FP	GTGGAGTAGGTCTGGAGGTGGACTCACGGCTGCTT
SDMguideC RP	AAGCAGCCGTGAGTCCACCTCCAGACCTACTCCAC
SDMguideD FP	CGGGGAAATCGACCGGACGCGGGAG
SDMguideD RP	CTCCCGCGTCCGGTCGATTTCCCCG

Guide RNA sequence

Guide A1	ACCGAGGTCCAGACCTACTCCACA
Guide A2	AAACTGTGGAGTAGGTCTGGACCT
Guide B1	ACCGTGCTTGGAGCGTCCGCCATG
Guide B2	AAACCATGGCGGACGCTCCAAGCA
Guide C1	ACCGCTCCAAGCAGCCGTGAGTCC
Guide C2	AAACGGACTCACGGCTGCTTGGAG
Guide D1	ACCGTGCGGGTTGCGGGGAAATCG
Guide D2	AAACCGATTTCCCCGCAACCCGCA

Analysis Primers

FPPRC1N-Terminus OutSide LHA1	TGCCAAACAAGGAAATGCCAGTAT
RPPRC1N-Terminus inside RHA1	CAACCCGCACCTTCTCCTCA
RPPRC1N-Terminus Outside RHA1	CCTTGATAGAGGAGATGCCTAC
mGFP (PRC1-N)FP	ATGAGCGTGAGCAAGGGCGA
mCherry(PRC1-N)FP	ATGGTGAGCAAGGGCGAGGA
mCherry(PRC1-N)RP	CGCACCTTCTCCTCATTCCGCCTCCTCCGCCCTTGTACAGCTCGTCCATGCC

Sequencing Primers

mcherryRPseqforPRC1	TGAACTCCTTGATGATGGCCATGT
PRC1 LHAFPseq	GTCACGGGCATGCGTGCACA
mGFP primer	GTCCTTAAGGAGTTCGTGACCG
