# Supplementary material for: Gradual compaction of the central spindle decreases its dynamicity in PRC1 and EB1 gene-edited cells
Source: Life Sci Alliance. 2021 Sep 27;4(12):e202101222. doi: 10.26508/lsa.202101222 (PMC8500333; doi:10.26508/lsa.202101222)
Supplement: Supplementary file 9 [file LSA-2021-01222_Supplemental_Data_2.pdf]

EB1 donor vector primers

EB1 FP left H arm	TCGGTACCCGGGGATCGGAAATAGGATCTCACTGCC
EB1 RP left H arm	CCTTGCTCACGCTCATCTTCTAAAGCATGGGAAGAAAAG
mGFP FP EB1	ATGAGCGTGAGCAAGGGCGA
mGFP RP EB1	TATACGTTCACTGCCATGCCATCCACCGCGCCTTCGCCGCCGCTGCCCGC GCCGCCGCTGCCGCCCGCCTGCGCCGCGTATCCGCCTCCCTTG
EB1 FP Right H arm	ATGGCAGTGAACGTATACTCAA
EB1 RP Right H arm	TCGACTCTAGAGGATCACCACACCGAGACTTTAAATCA

SDM primers for the PAM motifs

EB1 SDM guide A FP	GAACAGTTGTGCTCAGTTAAGAGAAATCTGCTG
EB1 SDM guide A RP	CAGCAGATTTCTCTTAACTGAGCACAACTGTTC
EB1 SDM guide B FP	GACATGACATGCTGGCTTGGATCAATGAGTCTC
EB1SDM guide B RP	GAGACTCATTGATCCAAGCCAGCATGTCATGTC
EB1 SDM guide C FP	GACATGACATGCTGGCTTGGATCAATGAGTCTC
EB1SDM guide C RP	GAGACTCATTGATCCAAGCCAGCATGTCATGTC
EB1 SDM guide D FP	CAGGTAAGAGAAATCTGCTTTATCATTTTTCTAGGAAAGC
EB1 SDM guide D RP	GGCTTTCCTAGAAAAATGATAAAGCAGATTTCTCTTACCTG

Guide RNA sequence

Guide A1	ACCGAAGATCGAACAGTTGTGCTC
Guide A2	AAACGAGCACAACTGTTCGATCTT
Guide B1	ACCGCTGCAGAGACTCATTGATCC
Guide B2	AAACGGATCAATGAGTCTCTGCAG
Guide C1	ACCGCTGCAGAGACTCATTGATCC
Guide C2	AAACGGATCAATGAGTCTCTGCAG
Guide D1	ACCGCTCAGGTAAGAGAAATCTGC
Guide D2	AAAC GCAGATTTCTCTTACCTGAG

Analysis Primers

EB1FPoutsideLHA	CTGTAAGGTCATTTGATACTGCC
EB1RPinsideRHA	GTCATGTCGACTTAGGTTATCAC
mGFP FP EB1	ATGAGCGTGAGCAAGGGCGA
mGFP RP EB1	TATACGTTCACTGCCATGCCATCCACCGCGCCTTCGCCGCCGCTGCCCGC GCCGCCGCTGCCGCCCGCCTGCGCCGCGTATCCGCCTCCCTTG

Sequencing primers

mGFPseq for EB	GTCCTTAAGGAGTTCGTGACCG
EB1 RHA reverse	CTCTGTGTGTGGCTTTGCAGTC

EB1-GFP Lenti viral Cloning Primers

Forward Primer	CCGCTCGAGATGGCAGTGAACGTATACTCA
Reverse Primer	CGCGGATCCCGCGTATCCGCCTCCCTTG

KIF4A-mGFP Lenti viral Cloning Primers

Forward Primer	CTCAAGCTTCGAATTATGAAGGAAGAGGTGAAGGGAAT
Reverse Primer	TAGAGTCGCGGGATCTTACTTGTACAGCTCGTCCATG

EGFP-CLASP1 Lenti viral Cloning Primers

Forward Primer	CTCAAGCTTCGAATTATGGTGAGCAAGGGCGAGGA
Reverse Primer	TAGAGTCGCGGGATCTTAGCTGTGCGTGGAGACATC
